# Quantitative Analysis of Mitochondrial DNA Heteroplasmy in Urinary Podocytes of Myoclonus Epilepsy With Ragged-Red Fibers Syndrome

**DOI:** 10.1016/j.ekir.2023.09.021

**Published:** 2023-09-17

**Authors:** Miwa Goto, Emi Sawanobori, Takeshi Inukai, Shuji Hirata, Tadashi Mabuchi

**Affiliations:** 1 Department of Pediatrics, Faculty of Medicine, University of Yamanashi, Shimokato, Chuo, Yamanashi, Japan; 2 National Hospital Organization, Kofu National Hospital, Tenjin, Kofu, Yamanashi, Japan; 3 Department of Obstetrics and Gynecology, Faculty of Medicine, University of Yamanashi, Shimokato, Chuo, Yamanashi, Japan

**Keywords:** fragment analysis, heteroplasmy, mitochondrial diseases, proteinuria, standard curve, urinary podocytes

## Introduction

Mitochondria are the critical organelles to generate cellular energy through oxidative phosphorylation. Their functions are regulated by mitochondrial DNA (mtDNA) as well as nuclear DNA. Gene mutations can lead to mitochondrial dysfunction in cells or tissues and result in mitochondrial diseases.^1^

In diseases caused by mtDNA mutations, manifestations are evident in tissues with high-energy demands and composed of postmitotic cells, which potentially accumulate mtDNA mutations.^2^

The kidneys are commonly involved in mitochondrial diseases.^3^^,^S1 Known mitochondrial nephropathies include tubular dysfunction with damage to tubular epithelial cells and focal segmental glomerulosclerosis with podocyte damages.S2 Both cells require high amounts of energy, and podocytes also exhibit the characteristics of postmitotic cells. Given that mtDNA has multiple copies in a single cell, most pathogenic mutations coexist with wild-type mtDNA, which is termed as heteroplasmy.^4^ The heteroplasmy degree varies widely among tissues, and mitochondrial defects are observed only when the percentage of pathogenic mtDNA surpasses the tissue-specific thresholds, which usually range from 60% to 90%.^5^ Therefore, the target of diagnosis of mitochondrial nephropathy must involve the pathological mitochondrial abnormalities as well as qualitative and quantitative abnormalities of mtDNA in cells forming nephrons.

We recently encountered a patient with myoclonus epilepsy with ragged-red fibers caused by an m.15923A>G mutation in tRNA^Thr^ genes in mtDNA^6^ who had glomerular proteinuria since childhood. Although we considered performing a pathological assessment of the kidneys to reveal the association between proteinuria and myoclonus epilepsy with ragged-red fibers, renal biopsy was difficult to perform because of the patient’s conditions. Here, the aim was to elucidate whether qualitative and quantitative mtDNA abnormalities were present in the podocytes excreted in the urine.S3 Then, we analyzed the accurate heteroplasmy level of mutant DNA by fragment analysis calibrated by use of the standard curve.

This study was approved by the ethics committee of the Faculty of Medicine, University of Yamanashi. Informed consent to treatment and to the publication of the results was obtained from the patient’s guardian.

## Results

### A15923G mtDNA Mutation in Both Urinary Podocytes and Blood Cells

DNA fragments (1499 bp) encompassing nucleotide position 15923 were amplified by polymerase chain reaction using DNAs extracted from urinary podocytes and blood cells as templates. We then confirmed the presence of the A15923G mutation in both urinary podocytes and blood cells (data not shown). The patient`s clinical course and laboratory data are described in the Supplementary Methods and Supplementary Figure S1. The experimental protocols and immunofluorescence staining images of urinary podocytes are also available in the Supplementary Methods and Supplementary Figure S2.

### Precise Analysis of mtDNA Heteroplasmy in Urinary Podocytes

**Figure 1:**
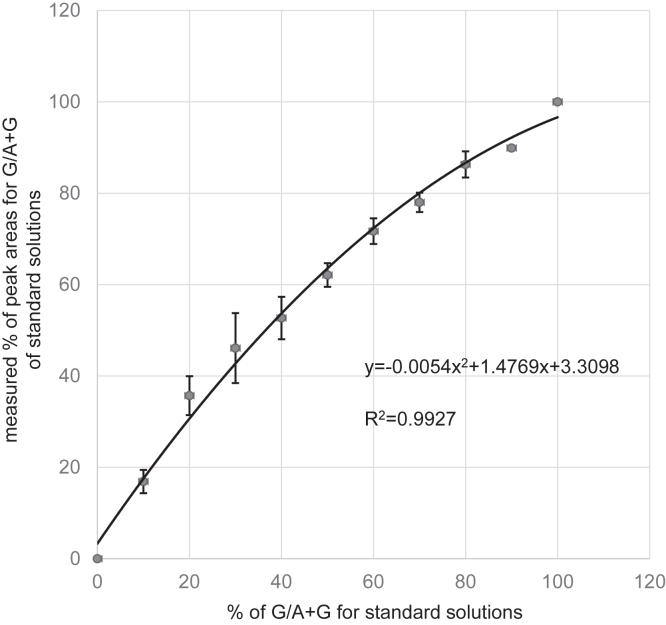
Standard curve. The standard curve was not linear and was instead upward with respect to the calculated line.

The level of heteroplasmy was determined by fragment analysis. Heteroplasmy levels of m.15923A>G were 72% in urinary podocytes and 29% in blood cells as calibrated by the standard curve (Figure 1), whereas the heteroplasmy determined as area ratios conventionally used were 82% and 41%, respectively.

## Discussion

Many podocytes were observed in the patient’s urine, suggesting that podocytes were the major site of dysfunction. Clinically, mitochondrial nephropathy was suspected. However, a renal biopsy was not performed due to her condition. Instead, we conducted fragment analysis to examine the heteroplasmy loads of the mtDNA mutation in urinary podocytes.

Recently, pyrosequencing, quantitative polymerase chain reaction, and fragment analysis have been performed to quantify heteroplasmy levels of mutant mtDNA.S4–S6 Fragment analysis is easy to perform, and mutation rates have been determined primarily as ratios of peak heights or peak areas. However, differences in the fluorescence intensities of each dye labeled, and incorporation rates of labeled nucleotides into extending polymerase chain reaction products cause divergence of results from the correct values. Thus, we constructed a standard curve to more accurately analyze the heteroplasmy levels of the samples. The analyzed percentages (y-axis) were not identical to the standard percentages (x-axis). In this study, the heteroplasmy levels were 72% in urinary podocytes and 29% in the blood. These levels, when determined without the collection as the conventional area ratios, were 82% and 41%, respectively, varying from those determined using the standard curve. The result shows the importance of using the standard curve for accurate analysis.

In this patient, podocytes with 72% heteroplasmy were found in the urine. Based on the 3 case reports, including this patient,^6^, ^7^, ^8^ the threshold for manifestation would be between 33%^8^ and almost 100%^6^ in muscles for this mutation. Taken together, our results strongly suggest that the accumulation of mutant mtDNA cause podocyte damage, resulting in proteinuria in our patient. Lin *et al.* reported that mitochondrial protein synthesis is not completely inhibited *in vitro* even when this mutation accounts for 100%.^6^ Therefore, the renal dysfunction in this patient may only be proteinuria, even though the heteroplasmy level was 72%.

The diagnosis of mitochondrial disease should be assessed by mtDNA heteroplasmy levels in clinically affected tissues. Renal biopsy is highly invasive, particularly in children, and is not always feasible. It was previously reported that urinary epithelial cells may be used to diagnose mitochondrial disease, such as the 3243A>G mutation, because the heteroplasmy load in epithelial cells were comparable to that in the muscle.^9^ However, urinary epithelial cells are not always included in nephron component cells; in this case, they are not suitable to diagnose mitochondrial nephropathy. Here, we focused on podocytes excreted in the patient’s urine for the diagnosis of kidney injury caused by mitochondrial disease. We demonstrated the utility of urinary podocytes specifically isolated using immunoprecipitation as a noninvasive method of diagnosis for patients with suspected mitochondrial nephropathy.

## Disclosure

All the authors declared no competing interests.

## Supplementary Material

**Supplementary Methods.**

**Supplementary Reference.**

**Figure S1.** Clinical course and laboratory data.

**Figure S2.** Immunofluorescence staining images of urinary podocytes before and after isolation by using magnetic beads.

## References

[1] Wallace D.G. (2013). A mitochondrial bioenergetics etiology of disease. J Clin Invest.

[2] Tuppen H.A.L., Blakely E.L., Turnbull D.M., Taylor R.W. (2010). Mitochondrial DNA mutations and human disease. Biochim Biophys Acta.

[3] Cavero T., Rabasco C., Molero A. (2015). When should a nephrologist suspect a mitochondrial disease?. Nefrologia.

[4] Nissanka N., Moraes C.T. (2020). Mitochondrial DNA heteroplasmy in disease and targeted nuclease-based therapeutic approaches. EMBO Rep.

[5] Rossignol R., Faustin B., Rocher C., Malgat M., Mazat J.P., Letellier T. (2003). Mitochondrial threshold effects. Biochem J.

[6] Lin H., Miyauchi K., Harada T. (2018). CO_2_-sensitive tRNA modification associated with human mitochondrial disease. Nat Commun.

[7] del Mar O’Callaghan M., Emperador S., López-Gallardo E. (2012). New mitochondrial DNA mutations in tRNA associated with three severe encephalopamyopathic phenotypes: neonatal, infantile, and childhood onset. Neurogenetics.

[8] Kärppä M., Kytövuori L., Saari M., Majamaa K. (2018). Mutation m.15923A>G in the MT-TT gene causes mild myopathy - case report of an adult-onset phenotype. BMC Neurol.

[9] McDonnell M.T., Schaefer A.M., Blakely E.L. (2004). Noninvasive diagnosis of the 3243A>G mitochondrial DNA mutation using urinary epithelial cells. Eur J Hum Genet.

