# Supplementary material for: Quantitative Analysis of Mitochondrial DNA Heteroplasmy in Urinary Podocytes of Myoclonus Epilepsy With Ragged-Red Fibers Syndrome
Source: Kidney Int Rep. 2023 Sep 17;8(12):2830–2. doi: 10.1016/j.ekir.2023.09.021 (PMC10719592; doi:10.1016/j.ekir.2023.09.021)
Supplement: Supplementary File (PDF) [file mmc1.pdf]

Supplementary File (PDF)

Supplementary Methods

Case report

The patient was an 18-year-old Japanese girl with no family history of consanguineous marriage. She was born at 39 weeks and weighed 3,070 g. Her gestational and postpartum courses were uneventful. Except for diabetes mellitus in her maternal grandfather, the patient's family history was unremarkable. During infancy, she did not exhibit any signs of hearing loss, and her motor development was normal. The course to the diagnosis of mitochondrial disease was described previously [6] . Briefly, she developed hearing loss in both ears at the age of 6, and at 10 to 11 years old, she had a short stature (3.4 standard deviations) and was noted to have lesions in the cerebellum, basal ganglia, and bilateral dentate nuclei of the hypothalamus on cranial MRI. At the same time, she was diagnosed with epilepsy and retinitis pigmentosa. Mitochondrial disease was then suspected due to the elevated lactate/pyruvate ratio in the blood and spinal fluid and a muscle biopsy was performed at the age of 12 years. Whole mtDNA sequencing of muscle tissue revealed an m.15293A>G mutation in an almost homoplasmic state; together with the results of pathological analysis of the muscle, this confirmed the diagnosis of mitochondrial disease.

Proteinuria was first detected at the age of 10 years. Since then, proteinuria (urinary total protein/creatinine [UTP/Cr] ratio of 0.5 to 1.0 g/g·Cr) had persisted. Although her renal function remained unchanged during the course of the disease, severe proteinuria (UTP/Cr ratio of ≥1.0 g/g·Cr) had persisted after the age of 16 years. Based on routine tests during this period, no signs of tubular disorder were observed. In the spot urine samples, the UTP/Cr ratio was 1.15 g/g·Cr, and the urinary albumin/creatinine ratio was 973.8 mg/g·Cr, both being elevated at the age of 18. In addition, podocyturia was detected, suggesting that podocyte damage (Supplementary Figure S1).

Isolation of podocytes from urinary sediments

After fresh spot urine samples were collected and centrifuged, the precipitates were suspended in phosphate-buffered saline. The samples were mixed with a rotator with an anti-podocyte antibody (mouse monoclonal antibody to human epithelial cells [MAB430]; Millipore, Billerica, MA) bound to magnetic beads (Dynabease®Goat anti-Mouse IgG: Invitrogen, Waltham, MA). Thereafter, urinary podocytes were harvested with a magnetic separator and confirmed by immunofluorescence staining (Supplementary Figure S2).

DNA extraction, polymerase chain reaction (PCR), and nucleotide sequencing

Total DNA was extracted from either the urinary podocytes (ca. 1000 to 3000 podocytes) or whole blood (30 μL) using the Kaneka DNA Easy Extraction Kit version 2 (Kaneka, Takasago, Hyogo, Japan).

PCR was performed to amplify the 1,499 base-pair (bp) DNA fragment using extracted DNAs as templates. Primers were set at encompassing position 15923 (the primer set 1: mtDNA15923U1: GCGACCCAGACAATTATACCC; mtDNA15923D1: GTTAAAAGTGCATACCGCCAA), and Kaneka High-speed DNA polymerase (Kaneka) or Phusion Hot Start Flex DNA polymerase (NEB, Ipswich, MA) was used.

The purified DNA fragments were directly sequenced using two primers (mtDNA15923U2: TTGACTCCTAGCCGCAGACCT; mtDNA15923D2: TACATAGCGGTTGTTGATGGG) in addition to the primer set 1.

Construction of calibration curve

PCR products from whole-blood DNA that were not cleaved with *Acc*I were extracted from the gel and purified to be used as wild-type mtDNA. Next, another PCR was performed using primer set 2 (mtDNA15923U3: CGGAATTCAACCTGAAACATCGGCATTATCCTC; mtDNA15923D3: GCCCTAGGCTGGCACGAAATTGACCAACCCTG) wherein DNA from urinary podocytes served as a template. The PCR products were cloned into the plasmid pUC118. The plasmids that had the *Acc*I site were selected as templates for PCR using primer set 1. The obtained products were purified and used as mutant mtDNA. Using wild-type and mutant DNA fragments, 11 standard solutions were prepared at serial proportions of mutant mtDNA from 0% to 100% in 10% increments and were used for fragment analysis to construct the standard curve each time for the assay. The standard curve was generated by plotting the percentages of peak areas for G/A+G, and percentages of the peak areas were measured for G/A+G obtained by fragment analysis on the x- and y-axes, respectively (Figure 1). The second-order polynomial approximation and coefficient of determination for the calibration curve were calculated using Microsoft Excel.

Fragment analysis

Single base primer extension reaction was performed with a thermal cycler using the Snapshot Multiplex kit (Thermo Fisher Scientific, Waltham, MA). The reaction products were

purified using the ExoSAP-IT PCR Product Cleanup kit (Thermo Fisher Scientific), and fragment analysis was performed. Three independent analyses were performed for urinary podocytes and blood cells in triplicate.

Supplementary References

S1. Kisler JE, Whittaker RG, McFarland R. Mitochondrial diseases in childhood: a clinical approach to investigation and management. Dev Med Child Neurol. 2010;52:422-433. DOI: 10.1111/j.1469-8749.2009.03605.x

S2. Govers LP, Toka HR, Hariri A et al. Mitochondrial DNA mutations in renal disease: an overview. Pediatr Nephrol. 2021;36:9-17. DOI: 10.1007/s00467-019-04404-6

S3. Whittaker RG, Blackwood JK, Alston CL et al. Urine heteroplasmy is the best predictor of clinical outcome in the m.3243A>G mtDNA mutation. Neurology. 2009;72:568-569. DOI: 10.1212/01.wnl.0000342121.91336.4d

S4. Singh R, Ellard S, Hattersley A et al. Rapid and sensitive real-time polymerase chain reaction method for detection and quantification of 3243A>G mitochondial point mutation. J Mol Diagn. 2006;8:225-229. DOI: 10.2353/jmoldx.2006.050067

S5. Sosa MX, Sivakumar IKA, Maragh S et al. Next-generation sequencing of human mitochondrial reference genomes uncovers high heteroplasmy frequency. PLoS Comput Biol. 2012 8:e1002737. DOI: 10.1371/journal.pcbi.1002737

S6. Cassandrini D, Calevo MG, Tessa A et al. A new method for analysis of mitochondrial DNA point mutations and assess levels of heteroplasmy. Biochem Biophys Res Commun. 2006;342:387-393. DOI: 10.1016/j.bbrc.2006.01.152

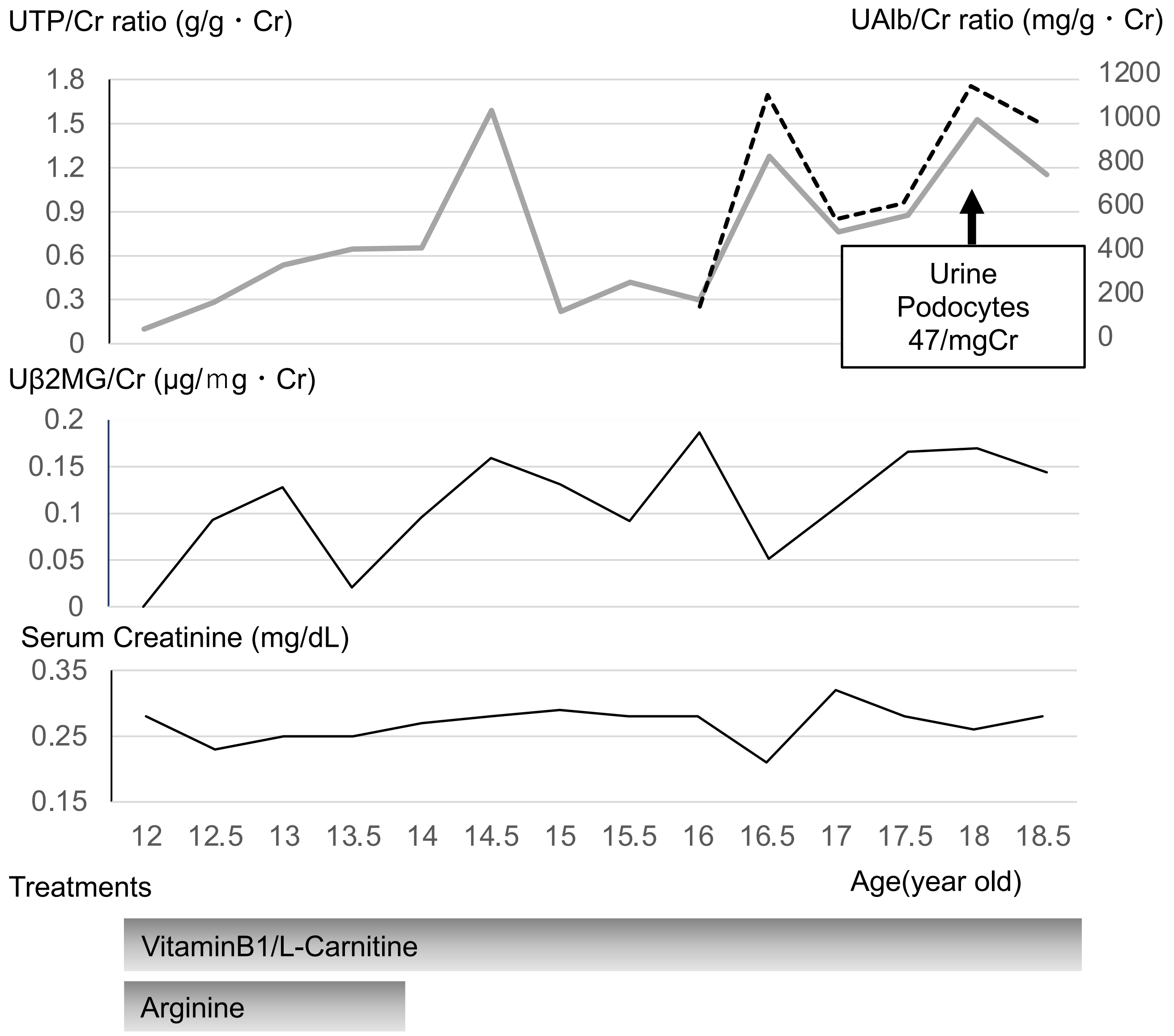

Supplementary Figure S1.

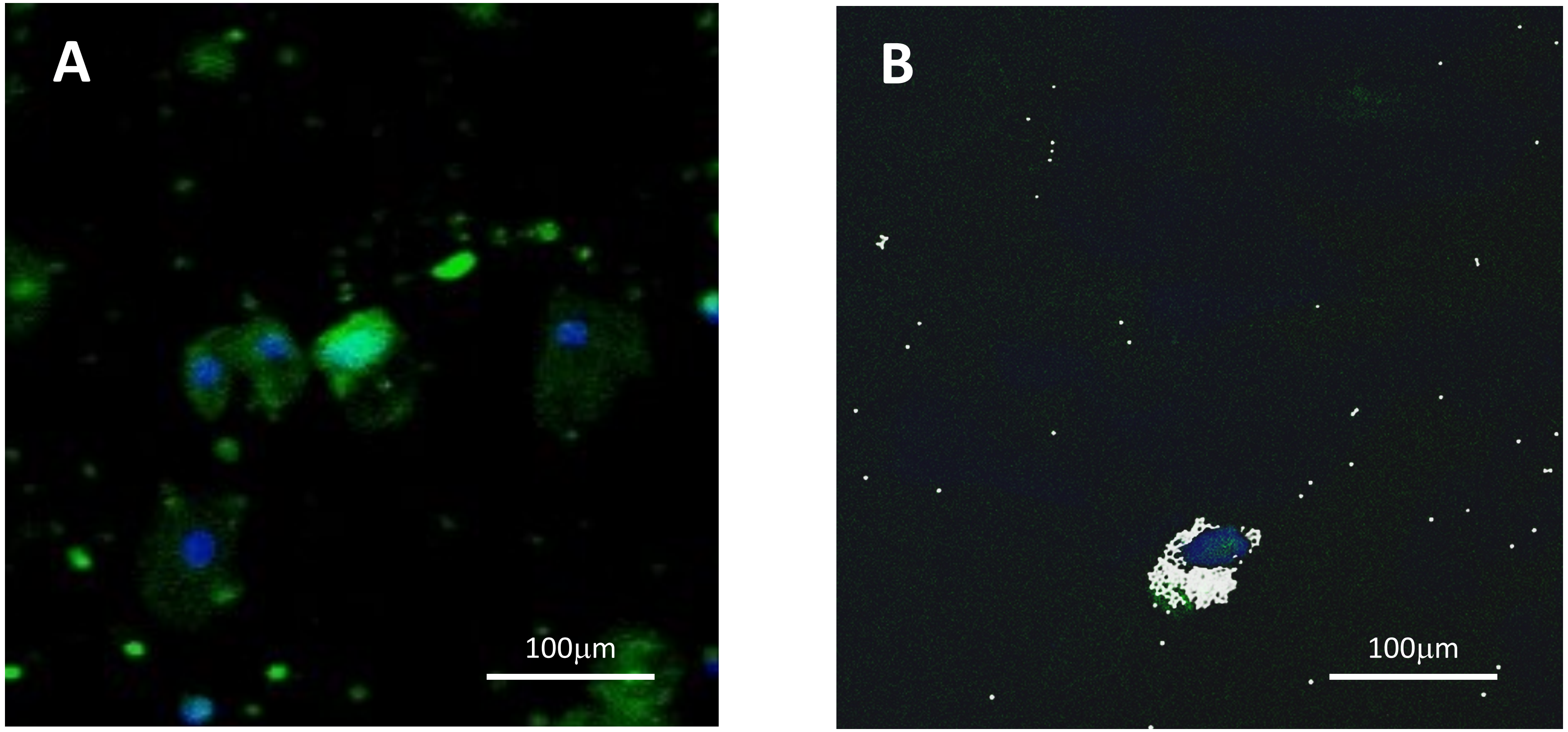

Supplementary Figure S2.

Supplementary Figure S1. Clinical course and Laboratory data

UTP/Cr ratio, urinary total protein/creatinine ratio (solid line); UAlb/Cr ratio, urinary albumin/creatinine ratio (dashed lines). Uβ2MG/Cr , urine beta2 microglobulin/creatinine ratio: Urinary podocytes number at age 18 are shown in the graph.

Supplementary Figure S2. Immunofluorescence staining images of urinary podocytes before (A) and after (B) isolation by using magnetic beads.

Podocyte specific marker podocalyxin (green) , cell nucleus stained with 4',6-diamidino-2-phenylindole (DAPI; blue) are shown. Also, magnetic beads are shown as white to show clearly. Scale bars, 100 μm. Shedding vesicles derived from podocytes were rarely observed and were negligible after isolation.
